# Identifying potential novel widespread determinants of bacterial pathogenicity using phylogenetic-based orthology analysis

**DOI:** 10.3389/fmicb.2025.1494490

**Published:** 2025-05-01

**Authors:** Sara Ribeiro, Karine Alves, Julien Nourikyan, Jean-Pierre Lavergne, Simon de Bernard, Laurent Buffat

**Affiliations:** ^1^AltraBio SAS, Lyon, France; ^2^Molecular Microbiology and Structural Biochemistry, Université de Lyon, CNRS, Lyon, France

**Keywords:** bacterial pathogenicity, orthology analysis, comparative proteomics, therapeutic targets, diagnostic methods

## Abstract

**Introduction:**

The global rise in antibiotic resistance and emergence of new bacterial pathogens pose a significant threat to public health. Novel approaches to uncover potential novel diagnostic and therapeutic targets for these pathogens are needed.

**Methods:**

In this study, we conducted a large-scale, phylogenetic-based orthology analysis (OA) to compare the proteomes of pathogenic to humans (HP) and non-pathogenic to humans (NHP) bacterial strains across 734 strains from 514 species and 91 families.

**Results:**

Using a dedicated workflow, we identified 4,383 hierarchical orthologous groups (HOGs) significantly associated with the HP label, many of which are linked to critical factors such as stress tolerance, metabolic versatility, and antibiotic resistance. Both known virulence factors (VFs) and potential novel widespread pathogenicity determinants were uncovered, supported by both statistical testing and complementary protein domain analysis.

**Discussion:**

By integrating curated strain-level pathogenicity annotations from BacSPaD with phylogeny-based OA, we introduce a novel approach and provide a novel resource for bacterial pathogenicity research.

## 1 Introduction

The growing threat of antibiotic-resistant bacterial pathogens emphasizes the critical need to discover potential new determinants of pathogenicity for advancing therapeutic strategies. Databases such as the Virulence Factor Database (VFDB) catalog experimentally validated virulence factors (VFs) of pathogenic to humans (HP) bacteria (Liu et al., 2022), but the continual emergence of new pathogens underscores the need for methods that can uncover potential novel widespread pathogenicity determinants not yet captured in existing resources to be prioritized for future research.

Efforts to computationally identify genes, proteins, or domains associated with bacterial pathogenicity have been conducted in both plant- and human-associated bacteria. Studies by Studholme et al. (2005) and te Molder et al. (2021) have developed pathogenicity prediction tools and assessed protein domain-based analysis respectively for plant pathogens, while research by Naor-Hoffmann et al. (2022), Lobb et al. (2021), and Cosentino et al. (2013) have developed pathogenicity prediction tools for human pathogens. These studies have provided broad-spectrum insights into pathogenic traits. However, they primarily relied on protein families defined through sequence-based comparisons, which may overlook deeper evolutionary relationships (Altenhoff et al., 2019; Emms and Kelly, 2019). This is particularly important to be considered in bacteria, where processes such as horizontal gene transfer and gene duplications can introduce functionally similar proteins from different species or result in multiple copies of a gene within the same organism. Both mechanisms contribute to genetic diversity, even among proteins with similar functions, complicating sequence-based comparisons and the identification of potential novel conserved pathogenicity determinants. This complexity necessitates advanced computational approaches that consider both sequence and evolutionary context, such as orthology analysis (OA), which group proteins by shared ancestry (Altenhoff et al., 2019; Emms and Kelly, 2019).

Several studies have combined comparative genomics and proteomics with orthology-based analysis in bacteria to uncover conserved genes and identify potential novel therapeutic targets. For example, Aswal et al. (2020) utilized cross-genus proteomic analysis to identify granuloma-associated proteins in *Yersinia pseudotuberculosis*. Similarly, studies on *Pseudomonas aeruginosa* (Sood et al., 2019) and *Corynebacterium* species during a diphtheria outbreak (Xiaoli et al., 2023) focused on genomic plasticity and positive selection in core genes associated with virulence. In addition, Cuadrat et al. (2014) investigated conserved genes across diverse organisms, including protozoa, prokaryotes, and model eukaryotes, with a focus on gene function conservation. The latter relied on model organisms to provide a comparative framework.

However, the algorithms applied for OA in these studies lacked phylogenetic context, limiting their ability to reliably uncover potential novel pathogenicity determinants. An approach based on hierarchical orthogroups (HOGs), on the other hand, could overcome this challenge by accounting for both speciation events (evolutionary divergence) and gene duplications. This generally allows for a more accurate inference of protein function across species (Emms and Kelly, 2019). Additionally, the mentioned studies have a narrow focus, concentrating on specific bacterial species, genus, pathogenic pathways or core genes. The previous lack of a curated database for pathogenicity annotations likely constrained these studies by limiting the inclusion of a broader diversity of bacterial strains, species, and families. This gap may have also compelled researchers to rely on erroneous assumptions regarding their pathogenicity annotations (Ribeiro et al., 2024).

In this study, we introduce a novel approach for identifying potential new and widespread bacterial pathogenicity determinants by applying a phylogenetic-based OA across a wide spectrum of bacterial taxa coupled with rigorously curated strain-level pathogenicity annotations. Our method prioritizes genome completeness and precise annotations. By analyzing 734 strains from 514 species with these annotations, we could redefine bacterial proteomes with HOGs across 23% of currently known bacterial species able to infect humans and uncover potential novel widespread pathogenicity determinants, including most common pathogens, as demonstrated by a comparison with a comprehensive list of human-associated pathogens (Bartlett et al., 2022) and the FDA-ARGOS Wanted Organism list (Sichtig et al., 2019). This ensured the inclusion of clinically relevant species, genera, and families across 12 major bacterial phyla. Through the integration of strain-level pathogenicity data from the Bacterial Strains’ Pathogenicity Database (BacSPaD) (Ribeiro et al., 2024) and a dedicated workflow using phylogenetic-based OA, we identified 4,383 HOGs associated with HP strains, highlighting both known VFs and potential novel widespread ones. These lists coupled with highlighted candidates, the resulting approach, framework and remaining HOGs data may provide important guidance for future bacterial pathogenicity research.

## 2 Materials and methods

### 2.1 Data acquisition and statistics-based selection

An initial dataset of annotations for high-quality and complete genome sequences labeled according to their pathogenicity to humans was retrieved from the BacSPaD database (Ribeiro et al., 2024)^1^. This dataset is also available in Zenodo^2^ for reference. The HP and non-pathogenic to humans (NHP) strain categories in this database were defined based on documented associations with infectious processes, using a rule-based framework that integrates reliable metadata from medical and experimental observations. Each entry was rigorously and manually curated to ensure accuracy.

Genomes were then subject to a rigorous filtering and selection process to ensure a balance between data quantity and quality for OA, while ensuring representativity for each species. This process aimed to minimize the impact of potential assembly artifacts, contamination, and annotation errors.

Firstly, genomes were filtered to retain only those with a CheckM (version 1.1.6; Parks et al., 2015) completeness score over 95% and for which the corresponding proteomes contained at least 500 proteins.

Secondly, species were categorized based on the number of available genomes, and for species with more than two genomes, a score-based selection process was applied to select representative genomes for each species. This process involved computing three z-scores for each, based on the number of protein-coding sequences, CheckM completeness, and CheckM contamination (inverted, with lower contamination being better) within its species group. The final score for each genome was calculated as the mean of these z-scores, providing a single metric to assess overall genome quality and completeness. Thereafter, genomes were ranked according to their scores. For species containing uniformly labeled genomes (either HP or NHP), the top two genomes were directly selected based on their final scores. For species containing mixed labeled genomes (HP and NHP, based on manual curation from the BacSPaD database, as previously mentioned in section 2.1), the top-scoring genome within each pathogenicity group was selected based on quality metrics (completeness, contamination, and number of protein-coding sequences). To further evaluate the taxonomical representativeness of both the OA and statistical analysis datasets, we compared it with the HP bacteria listed by Bartlett et al. (2022). This comparison was performed at species, genus, and family levels. Coverage percentages were calculated, and Venn diagrams depicting overlaps were generated. In addition, we assessed the number of the most common pathogens included in our dataset using the FDA-ARGOS Wanted Organism List as reference.

### 2.2 Inference of HOGs using OrthoFinder for phylogenetic-based OA

HOGs were inferred using OrthoFinder v2.5.5 (Emms and Kelly, 2019), which delineates orthogroups by combining sequence similarity with phylogenetic relationships. The phylogeny used in this analysis is based on the NCBI taxonomy framework, which OrthoFinder utilizes to construct species trees and infer hierarchical orthogroups. The phylogenetic distance in OrthoFinder was calculated using a species tree inferred from concatenated alignments of single-copy orthologs, following OrthoFinder’s built-in pipeline (Emms and Kelly, 2019). DIAMOND (Buchfink et al., 2015) was used for rapid, all-versus-all protein sequence comparisons, with scores normalized based on sequence length and phylogenetic distance.

### 2.3 Data processing, statistical analysis, and clustering

HOG data was converted into a binary matrix indicating the presence or absence of each HOG across the strains. The ones present in fewer than three strains were excluded from further analysis. Then, manually curated pathogenicity annotations from the BacSPaD database (Ribeiro et al., 2024) were associated with the corresponding proteomes, and each HOG was named according to the its most frequent protein name, as determined by a majority vote.

To identify HOGs significantly associated with either the HP or NHP label, we applied a two-sided Fisher’s exact test. This test, which detects significant associations in both directions, offers a thorough and cautious evaluation, making it particularly well-suited for identifying potential HP-related HOGs. Following Benjamini-Hochberg correction for multiple testing, HOGs with an FDR-adjusted *p*-value < 0.05 were subsequently ranked based on the number of HP and NHP strains in which they were present, as well as their FDR values.

To ensure the selection of a biologically and statistically meaningful subset of HOGs for further exploration, we performed an inflection analysis on the combined ranking scores of all significant HOGs. This included both the application of the *kneedle* algorithm (Satopaa et al., 2011) for global inflection detection and a first derivative (slope) analysis of the ranked scores to capture local transitions for the top 500 HOGs.

Hierarchical clustering was then applied to the top ranked HOGs significantly associated with the HP label based on the threshold from the inflection analysis and respective strains using Euclidean distance and complete linkage to reveal patterns of association with the HP strains. For this, we used a binary presence/absence matrix of the top 100 HOGs most significantly associated with HP, where each cell was assigned a value of 1 (presence) or 0 (absence). Euclidean distances were computed both between rows (strains) and columns (HOGs) based on these binary profiles, and hierarchical clustering was performed using complete linkage. In this context, the Euclidean distance between two strains reflects the number of HOGs for which their presence/absence status differs; likewise, the distance between two HOGs reflects the number of strains in which their presence/absence differs. To determine the optimal number of HOG clusters, we performed silhouette analysis on the hierarchical clustering results (complete linkage, Euclidean distance). In addition, strains were clustered within each pathogenicity or family group depending on the analysis, and the ones lacking significant HOGs associated with the HP label, hereafter referred to as “significant HOGs to HP” were manually ordered based on their family classification.

## 3 Results

### 3.1 Dataset overview and genome selection

**FIGURE 1:**
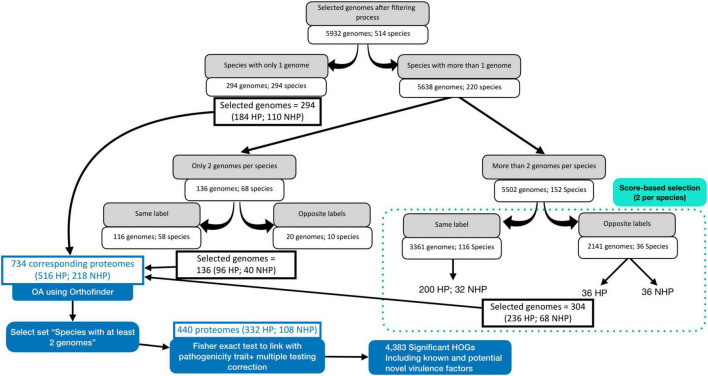
Workflow for selecting bacterial genomes and corresponding proteomes for orthology analysis (OA) and statistical evaluation. The process details the filtering and selection steps, ensuring representative and high-quality data for accurate downstream analysis.

The initial dataset, obtained from the BacSPaD database (Ribeiro et al., 2024), comprised 5,992 genomes representing 532 species. Applying the quality filtering criteria (as described in section 2.1) reduced the dataset to 5,932 genomes across 514 species (Figure 1). CheckM completeness-based filtering excluded 32 genomes, while the filtering step for proteomes with less than 500 proteins excluded 28 genomes. In total, 46 species of the 514 species retained (9%) contained both HP and NHP labeled genomes. For these mixed-labeled species with more than two available genomes, we selected one representative genome per label (HP and NHP) based on quality scores. For uniquely labeled species with more than two genomes, the top two genomes were selected using the same quality criteria. Species represented by exactly two genomes or by a single genome were all retained without further selection. This process resulted in a final OA dataset of 734 genomes, including 516 HP and 218 NHP strains. The curated BacSPaD labels were preserved throughout, and the selection procedure ensured that highest-quality genomes for each species were selected, thereby reducing technical noise while maintaining biological relevance. The corresponding proteomes, directly derived from these genomes selected from BacSPaD, were downloaded from the BV-BRC (Olson et al., 2023) FTP site to then be analyzed by OrthoFinder.

A comparison of this OA dataset with HP bacteria listed by Bartlett et al. (2022) revealed the following coverage levels: At the species level, it overlaps with 241 species (16%) of Bartlett et al.’s 1,513 listed HP species, with an effective coverage of 23% as 131 novel species were included in our data in comparison with this list. At the genus level, 103 of 327 genera (32%) were represented, with an effective coverage of 36% as 24 novel genera were included. At the family level, the dataset overlaps with 69 of 131 families (53%), rising to 55% upon inclusion of 6 novel families. Corresponding Venn diagrams to each of these taxa are included in Supplementary Figures 1-3.

### 3.2 Overview on OA results and most relevant HOGs

In total, OrthoFinder successfully assigned 2,492,963 proteins—representing 95.5% of the total protein set—to 124,904 HOGs. Notably, half of all proteins were found in HOGs containing 351 or more proteins, concentrated within the largest 1,423 HOGs. Additionally, we identified 52 orthogroups common to all strains, primarily comprising ribosomal proteins and essential housekeeping genes, which reflects evolutionary conservation and the robustness of the HOG inference approach. These universally conserved HOGs validate the method’s ability to capture core biological signals, distinguishing conserved core proteins from those related to strain-specific or pathogenicity-related functions. Conversely, 52,322 groups were excluded as they were not present in at least three strains. This dataset, hereafter referred to as “OA set,” was thus refined to 72,582 HOGs (Table 1). Detailed taxonomic information of this set can be found in the first tab of Supplementary Table 1 (Excel file).

**TABLE 1:** Summary of the set used for the orthology analysis (OA) and its resulting number of hierarchical orthogroups (HOGs) after filtering for presence in multiple strains.

Selection	Proteomes		HP		NHP		HOGs
OA set	734		516		218		72,582
	**Genera**	**Species**	**Genera**	**Species**	**Genera**	**Species**	
	187	514	127	373	100	187	

The number of proteomes and their distribution across genera and species, along with the distribution of the number of HP (pathogenic to humans) and NHP (non-pathogenic to humans) is summarized.

It is important to note that although some HOGs share similar functional annotations due to conserved domains, they are distinguished based on their evolutionary origins, reflecting distinct speciation, duplication, or horizontal gene transfer events (Emms and Kelly, 2019). Proteins from such events may retain similar functions but differ in aspects such as sequence, regulation, or structure, justifying their separation into distinct HOGs (Emms and Kelly, 2019).

Furthermore, annotations containing “@” [e.g., “3-ketoacyl-CoA thiolase (EC 2.3.1.16) @ Acetyl-CoA acetyltransferase (EC 2.3.1.9)”] were part of the original functional annotation. These denote enzymes with interchangeable or synonymous names due to their shared or overlapping catalytic activities, as defined in standard biochemical databases such as KEGG (Kanehisa et al., 2023).

### 3.3 Identification of key HOGs linked to HP

To evaluate the association between HOG presence and HP label, we conducted a statistical analysis using a pre-processed dataset that included two proteomes per species, hereafter referred to as “statistical set” (Figure 1). This analysis encompassed 440 strains, comprising 332 HP and 108 NHP strains across 220 species and 90 genera. After filtering for HOGs present in at least three strains, 51,745 HOGs were retained from the initial 72,582. Detailed taxonomic information of this set can be found in the second tab of Supplementary Table 1 (Excel file).

A taxonomy comparison of this statistical set with the list of pathogenic bacteria capable of infecting humans by Bartlett et al. (2022) revealed the following coverage levels: at the species level, the statistical set overlaps with 157 species (10%) of Bartlett et al.’s 1,513 listed HP species, with an effective coverage of 12% upon inclusion of 32 novel species. At the genus level, 70 of 327 genera (21%) were represented, with an effective coverage of 23% when including 6 novel genera. At the family level, the test set overlaps with 51 of 131 families (39%), rising to 44% upon inclusion of 6 novel families. Corresponding Venn diagrams to each of these taxa are included in Supplementary Figures 4-6. In addition, we could verify that 25 out of the 26 most common pathogens from the FDA-ARGOS Wanted Organism List were covered in this statistical set.

The statistical analysis described in section 2.3 revealed that 8,181 HOGs were significantly associated with either HP or NHP strains, from which 4,383 HOGs were significantly associated with HP strains (Table 2).

**TABLE 2:** Summary of the set used for statistical analysis (“statistical set”) and corresponding results.

Selection	HOGs	Proteomes		HP		NHP		HOGs significantly associated with HP or NHP labels (FDR <0.05)	HOGs significantly associated with HP label (FDR <0.05)
Statistical set (2 proteomes per species)	51,745	440		332		108		8,181	4,383
		**Genera**	**Species**	**Genera**	**Species**	**Genera**	**Species**		
		90	220	76	189	77	38		

The total number of proteomes and their distribution across genera and species is presented for the selected hierarchical orthogroups (HOGs), along with the breakdown of HP (pathogenic to humans) and NHP (non-pathogenic to humans) proteomes. The last two columns present the number of statistically significant HOGs (FDR < 0.05) after statistical analysis on this set: those associated with either HP or NHP labels, and those exclusively associated with the HP label.

Subsequently, to prioritize the most relevant HOGs associated with HP strains, we ranked these significant HOGs based on three metrics: the number of strains identified as HP, the number of strains identified as NHP, and the FDR-adjusted *p*-value. HP counts were ranked in descending order to emphasize HOGs prevalent in HP strains, while NHP counts and FDR-adjusted *p*-values were ranked in ascending order to highlight HOGs less common in NHP strains and those with stronger statistical significance. The final ranking was calculated by summing the ranks across these metrics, with the resulting table of HOGs sorted by this combined rank. From this sorted table, the inflection plot of combined ranking scores (Supplementary Figure 7) showed a steep decline within the first few hundred HOGs. While the kneedle algorithm identified a global inflection at rank 2, a slope analysis revealed the most pronounced score declines occurred within the top 100–200 ranks after the first top 5. Thus, the top 20 and top 100 HOGs were selected for comparative analysis, focusing on those most likely to influence bacterial pathogenicity based on their combined statistical and biological significance.

The inspection of the top 20 HOGs revealed key proteins associated with HP strains (Table 3 and Supplementary Table 2), with 4 of these 20 HOGs representing experimentally validated VFs. Specifically, these are: Periplasmic nitrate reductase (EC 1.7.99.4), which generally enables bacterial survival under oxygen-deprived conditions and is frequently encountered within host environments (Van Alst et al., 2007); Isocitrate lyase (EC 4.1.3.1), the first enzyme in the glyoxylate shunt, allowing bacteria to metabolize fatty acids as an energy and carbon source when primary carbon sources are limited (McKinney et al., 2000); and efflux systems, including ABC transporters and RND efflux pumps, which contribute to antibiotic resistance and enhance bacterial fitness under antimicrobial pressure (Coyne et al., 2011) (Li et al., 2016).

**TABLE 3:** Top 20 hierarchical orthologous groups (HOGs) significantly associated with pathogenicity.

HOG name	No. of HP strains	No. of NHP strains	No. of species	No. of genus	Combined rank	Experimentally verified VF	Category
Methylcrotonyl-CoA carboxylase biotin-containing subunit (EC 6.4.1.4)	90	0	45	21	2037.5	No	Nutritional/metabolic
Methylglutaconyl-CoA hydratase (EC 4.2.1.18)	84	0	42	18	2,277	No	Nutritional/metabolic
3-hydroxyisobutyrate dehydrogenase (EC 1.1.1.31)	136	3	74	34	2,502	No	Nutritional/metabolic
Ribonuclease E inhibitor RraA	74	0	37	15	2,642	No	Regulation
Uncharacterized protein, similar to the N-terminal domain of Lon protease	74	0	37	15	2,642	No	Stress survival
3-ketoacyl-CoA thiolase (EC 2.3.1.16) @ Acetyl-CoA acetyltransferase (EC 2.3.1.9)	97	1	51	25	2,672	No	Nutritional/metabolic
Acyl-CoA dehydrogenase	72	0	36	16	2,742	No	Nutritional/metabolic
Isovaleryl-CoA dehydrogenase (EC 1.3.8.4)	70	0	35	16	2,840	No	Nutritional/metabolic
Arginyl-tRNA–protein transferase (EC 2.3.2.8)	70	0	35	14	2,840	No	Regulation
Periplasmic nitrate reductase (EC 1.7.99.4)	101	2	52	21	2915.5	Yes	Stress survival
Fatty acid hydroxylase family (carotene hydroxylase/sterol desaturase)	87	1	45	22	2980.5	No	Nutritional/metabolic
Polyhydroxyalkanoic acid synthase	68	0	34	15	3003.5	No	Nutritional/metabolic
Putative hemolysin	66	0	33	14	3108.5	No, but yes for hemolysin	Uncertain; for hemolysin: exotoxin
Acyl-CoA dehydrogenase	65	0	33	14	3,199	No	Nutritional/metabolic
Isocitrate lyase (EC 4.1.3.1)	115	4	60	29	3,316	Yes	Others
Efflux ABC transporter, permease/ATP-binding protein	100	3	52	27	3,318	Yes	Effector delivery system
RND efflux system, membrane fusion protein	63	0	34	18	3325.5	Yes	Nutritional/metabolic factor
Glucose-1-phosphate thymidylyltransferase/RmlA (EC 2.7.7.24)	63	0	36	17	3325.5	No	Nutritional/metabolic factor
Hypothetical protein	63	0	32	17	3325.5	No	No categorization possible
Glyoxalase family protein	63	0	33	17	3325.5	No	Nutritional/metabolic factor

HOG name: Name of the HOG’s most frequent protein by majority vote; No. of HP/NHP strains: Number of pathogenic to humans (HP) and non-pathogenic to humans (NHP) strains containing the HOG; No. of species/genus: Number of species and genus containing the HOG; Combined rank: Overall rank combining rank of enrichment in HP strains (high number is higher ranked), enrichment in NHP strains (low number is higher ranked), and False Discovery Rate (FDR) value (low value is higher ranked). Decimal values indicate ties between multiple HOGs for the same metric; Experimentally verified VF: Indicates whether the protein or its associated function has been experimentally verified as a virulence factor (VF) in bacterial pathogenicity; Category: Classification based on functional roles; HOGs are ordered by the Combined rank column, with lower values indicating higher prioritization.

In addition to these experimentally validated VFs, our analysis uncovered HOGs comprising proteins with strong associations to HP strains, including ones previously suggested in the literature to be linked to bacterial pathogenicity (but not yet experimentally validated). For instance, the enzyme 3-hydroxyisobutyrate dehydrogenase (EC 1.1.1.31) was present in 136 HP strains but nearly absent in NHP strains. This enzyme is involved in valine degradation, a metabolic pathway that may contribute to providing an alternative energy source during nutrient limitation within host environments (Shahri et al., 2024). Importantly, it belongs to the short-chain dehydrogenase/reductase (SDR) enzyme family, which has been associated with metabolic processes contributing to virulence and antimicrobial resistance in other bacterial pathogens (Shahri et al., 2024). Our results also highlight HOGs linked to metabolic pathways essential for bacterial adaptation within the host. Proteins involved in fatty acid and amino acid degradation, such as methylcrotonyl-CoA carboxylase and methylglutaconyl-CoA hydratase, were particularly prominent. These enzymes are integral to leucine degradation pathways, which may provide alternative energy sources during host colonization (Kaiser and Heinrichs, 2018). These enzymes were also previously associated with HP strains from *Pseudomonas aeruginosa* (Díaz-Pérez et al., 2004; Höschle et al., 2005). Glyoxalase family proteins were also prominent. These proteins help mitigate oxidative stress, and may facilitate bacterial survival within hostile host environments. In *Escherichia coli*, the glyoxalase system is essential for acid resistance and survival during starvation (Bankapalli et al., 2015).

### 3.4 Distribution patterns of top significant HOGs to HP

To analyze the distribution of top HOGs across taxonomic groups and extend to a broader spectrum of proteins, we expanded the analysis to include the top 100 significant HOGs to HP. This threshold was defined after inspection of the slope analysis (Supplementary Figure 7) and allowed for the identification of key patterns while maintaining a dataset size conducive to detailed clustering and functional interpretation. A presence/absence heatmap was generated, with strains grouped by pathogenicity (HP or NHP) and clustered within each group on strains containing at least one HOG. Several HOGs were exclusively present in HP strains and absent in NHP strains across various taxa (Supplementary Figure 8). To capture underlying structure in HOG distribution patterns, we redefined clusters using unsupervised hierarchical clustering, with silhouette analysis supporting an optimal partitioning at *k* = 13.

**FIGURE 2:**
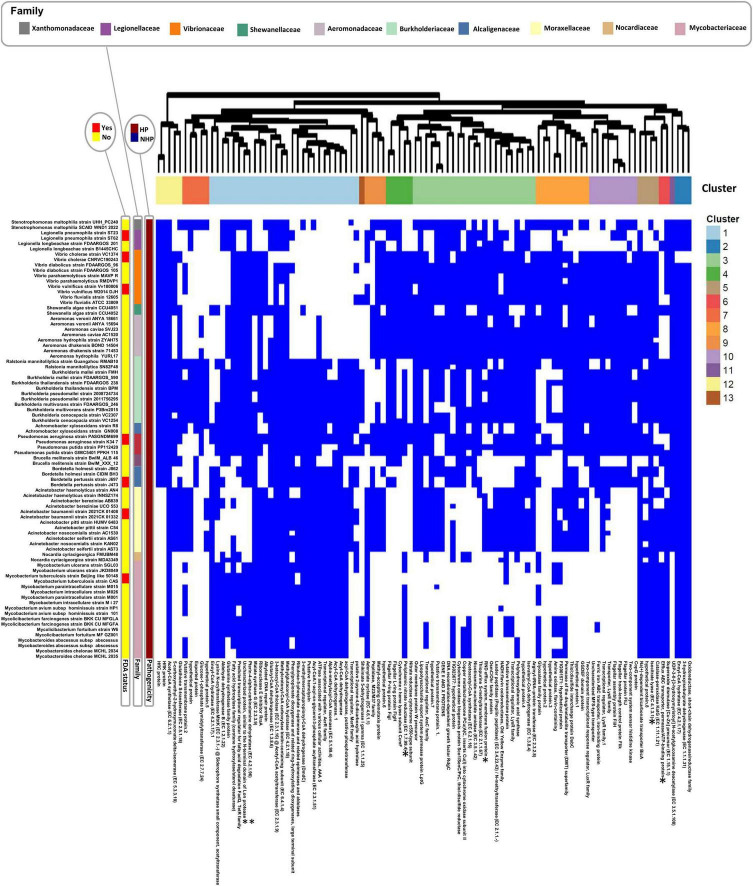
Presence/absence heatmap of the top 100 significant hierarchical orthogroups (HOGs) to HP for the set of strains with the highest HOG presence (82 strains in total). HOGs are listed below the heatmap, while strain names are displayed on the left. Hierarchical clustering was performed for both strains (within each pathogenicity group) and HOGs, using Euclidean distance as the metric and complete linkage as the clustering method. Distinct clusters resulting from hierarchical clustering of HOGs are annotated with different colors above the heatmap, highlighting groups with similar presence/absence patterns. Strains lacking these significant HOGs were manually grouped based on family classification. FDA-status annotations indicate whether a species is listed in the FDA-ARGOS Wanted Organism List (red: yes; yellow: no). Proteins sharing similar annotations are distinguished by a numerical suffix (e.g., “hypothetical protein.1”; “hypothetical protein.2”). *Experimentally validated VFs. **Protein similar to an experimentally validated VF.

From this set of top 100 significant HOGs, Figure 2 highlights the subset of strains with the highest HOG presence (located in the upper part of Supplementary Figure 8), comprising 82 strains in total.

For interpretability and conciseness, we focused on the five largest clusters—1, 3, 4, 8, and 10—which capture major trends in HOG distribution across HP and NHP strains. Cluster 4 was prioritized among similarly sized groups due to its inclusion of an experimentally validated VF. These can be broadly categorized by their protein functions and pathogen associations (Table 4).

**TABLE 4:** The five largest clusters from the top 100 significant HOGs identified in this study are summarized.

Cluster	Main functional category	Key proteins	Predominant pathogens	Notable observations
1	Metabolism, stress adaptation, regulation	Methylcrotonyl-CoA carboxylase, MbtK, Lon protease-like, RraA, AcrR, MerR	Burkholderia, Pseudomonas, Acinetobacter, Mycobacterium	Includes Lon protease-like protein (Lon protease is known virulence factor); linked to energy metabolism and adaptation.
3	Stress response, drug resistance, membrane integrity	RND efflux pump, AraC/LysR regulators, LptG, OMP-W precursor, peptidase/N-methyltransferase	Legionella, Vibrio, Burkholderia, Pseudomonas, Acinetobacter	Suggests roles in environmental survival; involves secretion and outer membrane integrity.
4	Anaerobic respiration, motility	Periplasmic nitrate reductase, CcmF, FlgH, FlgI, cytochrome c subunits	Vibrio, Burkholderia, Aeromonas, Pseudomonas	May support adaptation to low oxygen/biofilm conditions and host colonization.
8	Oxidative stress, signaling, biofilm regulation	Glyoxalase, DsbC, LuxR regulator, GGDEF domain protein	Vibrio, Aeromonas, Burkholderia, Acinetobacter	Suggests implication in stress signaling and quorum sensing via LuxR and GGDEF proteins.
10	Motility, iron acquisition, membrane transport	FliH/FliJ/FliK, LysR regulator, sensor histidine kinase, ferric iron ABC transporter	Vibrio, Aeromonas, Burkholderia, Pseudomonas, Bordetella	Highlights iron scavenging and motility as dual survival/virulence traits.

The Cluster column lists the numerical identifier for each; Main functional category: describes the primary biological process or pathway associated with each group; Key proteins: include representative proteins identified within each group; Predominant pathogens: highlight the main human pathogens which included these groups; notable observations: additional insights, such as the presence of hypothetical proteins, known virulence factors, or notable distribution patterns across strains.

Cluster 1 is centered on fatty acid and amino acid metabolism, as well as transcriptional regulation and stress adaptation. It includes previously discussed proteins of the top 20 HOGs such as methylcrotonyl-CoA carboxylase, methylglutaconyl-CoA hydratase, fatty acid hydroxylase, RraA, and a putative hemolysin. Additional proteins include a predicted fatty acid degradation regulator (FadQ), transcriptional regulators AcrR and MerR, and a glutamine synthetase family protein, all potentially involved in energy production and host adaptation. Notably, this cluster also contains Lysine N-acyltransferase MbtK, a siderophore biosynthesis enzyme in *Mycobacterium tuberculosis* (Harrison et al., 2006), and a protein similar to the N-terminal domain of Lon protease, a known VF in HP bacteria (*Kirthika et al., 2023*). This cluster is predominantly associated with *Burkholderia*, *Pseudomonas*, *Acinetobacter*, and *Mycobacterium*.

Cluster 3 highlights stress response, drug resistance, and membrane integrity, with several proteins previously described in the ranked table including the RND efflux system membrane fusion protein. Also present are regulatory and structural components such as AraC and LysR family transcriptional regulators, the outer membrane protein W precursor, and the lipopolysaccharide export protein LptG. A leader peptidase/N-methyltransferase supports potential roles in protein secretion and membrane assembly. This cluster is mainly associated with *Legionella*, *Vibrio, Burkholderia*, *Pseudomonas*, and Acinetobacter, reflecting its possible contribution to survival in stressful environments.

Cluster 4 focuses on anaerobic respiration and motility structures. It includes the periplasmic nitrate reductase, an experimentally verified VF linked to stress survival as previously mentioned, along with cytochrome c heme lyase (CcmF) and a nitrate reductase cytochrome c550-type subunit. These support survival in oxygen-limited or biofilm-associated environments. Structural components of the bacterial flagellum, including the flagellar L-ring (FlgH) and P-ring (FlgI) proteins, suggest roles in motility and host colonization. This cluster is predominantly found in *Vibrio, Burkholderia, Aeromonas, and Pseudomonas*.

Cluster 8 emphasizes oxidative stress regulation and signaling, and contains the previously discussed glyoxalase family protein, associated with biofilm formation and stress tolerance. The presence of DsbC, a thiol-disulfide isomerase important for outer membrane protein folding, and a LuxR-family response regulator, known to control quorum sensing and virulence gene expression in species like *Pseudomonas aeruginosa* and *Vibrio*, suggests a regulatory role. The co-occurrence of a GGDEF domain protein implies possible involvement in cyclic-di-GMP signaling, a key switch in biofilm and motility regulation. This cluster is primarily found in *Vibrio, Aeromonas, Burkholderia and Acinetobacter*.

Cluster 10 links motility, iron acquisition, and membrane transport, combining structural and regulatory proteins. It includes multiple flagellar assembly proteins (FliH, FliJ, FliK), a sensor histidine kinase, and a LysR family transcriptional regulator, all of which are important for motility, environmental sensing, and virulence regulation. The presence of a ferric iron ABC transporter, iron-binding protein highlights iron scavenging as a potential key adaptive trait in this cluster. This cluster is associated with *Vibrio* and *Aeromonas, Burkholderia, Pseudomonas, and Bordetella*, suggesting a role in both environmental sensing and host adaptation.

### 3.5 Family-level separation reveals HP-associated patterns

**FIGURE 3:**
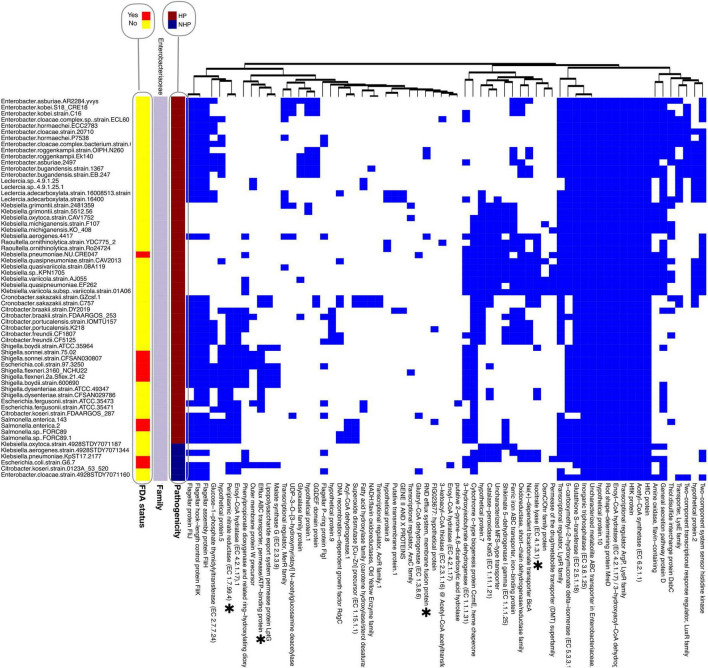
Presence/absence heatmap of the top significant hierarchical orthogroups (HOGs) found within the top 100 HOGs to HP for the Enterobacteriaceae family. To maintain consistency and comparability across analyses, the top 100 HOGs were retained for this family-based figure, with HOGs absent in all strains of this family omitted. HOGs are listed below the heatmap, while strain names are displayed on the left. Hierarchical clustering was performed for both strains (within each pathogenicity group) and HOGs, using Euclidean distance as the metric and complete linkage as the clustering method. HOGs were re-clustered for this figure based on presence/absence patterns in this subset, and thus the order does not reflect global cluster assignments. FDA-status annotations indicate whether a species is listed in the FDA-ARGOS Wanted Organism List (red: yes; yellow: no). Proteins sharing similar annotations are distinguished by a numerical suffix (e.g., “hypothetical protein.1”; “hypothetical protein.2”). *Experimentally validated VFs.

The separation of the top 100 HOGs by bacterial family, followed by clustering of strains with at least one HOG, reveals distinct distribution patterns that strongly correlate with the HP label. These patterns are especially evident in bacterial families containing both HP and NHP strains, such as in the Enterobacteriaceae family (Figure 3), the most represented family within the top 100 HOGs.

HOGs such as glucose-1-phosphate thymidylyltransferase, an enzyme involved in the biosynthesis of nucleotide sugars critical for cell wall and capsule formation (Xiao et al., 2021), were predominantly present in HP strains such as *Escherichia coli* (*E. coli*) and *Escherichia fergusonii* but absent in NHP strains within the same family. Catalase−peroxidase KatG, which plays a dual role in detoxifying reactive oxygen species and activating pro drugs like isoniazid, was also found in the HP strain of *E. coli* (*E. coli* strain G6 7), but not in the NHP strain (*E. coli* strain G976 372). The presence of these proteins in HP strains may corroborate their roles in supporting survival during infection and enhancing host-pathogen interactions.

Furthermore, the presence of a SDR enzyme in Corynebacteriaceae HP strains and absence in NHP strains from the same family (Supplementary Figure 9) suggests it as a potential novel VF for this family, as, to our knowledge, this is not yet described in literature. We also observe that Enoyl−CoA hydratase (EC 4.2.1.17) is consistently present in the HP strains of the Corynebacteriaceae family, but not for the NHP strains of the same family. This enzyme catalyzes a key step in fatty acid β-oxidation, aiding energy production and metabolic adaptation. Similarly, 3−hydroxyisobutyrate dehydrogenase (EC 1.1.1.31) and Oxidoreductase, short−chain dehydrogenase/reductase family are also shown to be present in various HP strains of this family, while absent in the corresponding NHP strains. As mentioned, these proteins may also be involved in energy production within host environments, by valine degradation (Shahri et al., 2024).

Shikimate 5-dehydrogenase I gamma (EC 1.1.1.25) and Glutaryl-CoA dehydrogenase (EC 1.3.8.6) are present in HP strains of both *K. pneumoniae* and the Corynebacteriaceae family, but absent in NHP strains of these species and family. Shikimate 5-dehydrogenase catalyzes the reduction of 3-dehydroshikimate to shikimate, a key step in the biosynthesis of essential aromatic compounds via the shikimate pathway. Glutaryl-CoA dehydrogenase oxidizes glutaryl-CoA to crotonyl-CoA, supporting energy production and biosynthetic processes through lysine and tryptophan degradation. Overall, these proteins are rarely found in NHP strains, with only two and one occurrences, respectively, across the entire data for the top 100 HOGs.

Interestingly, some families with prominent pathogens such as the Streptococcaceae family showed a scarce presence of these top 100 HOGs. When the analysis is expanded to include the top 200 HOGs, however, some HOGs are linked to HP strains within this family, as observed in Supplementary Figure 10. This suggests that highly conserved pathogenicity proteins are less prevalent in Streptococcaceae.

### 3.6 Coverage of known VFs

To compare our results with known VFs, we performed an overlap analysis with the VFDB database (Liu et al., 2022). The VFDB core dataset comprises 4,255 experimentally verified VFs. Using DIAMOND Blastp, we aligned their protein sequences with the ones from the 4,383 HOGs significantly associated with the HP label, applying stringent thresholds (*E*-value ≤ 10^−5^, identity ≥ 30% alignment).

From the 4,255 experimentally verified VFs from VFDB, 4,076 successfully mapped to the OA set, from which 4,033 successfully mapped to the statistical set.

From our set of 4,383 statistically significant HOGs to HP, 980 contained at least one experimentally verified VF recorded in VFDB, covering a total of 1,726 VFs. This represents about 43% of the experimentally verified VFs which mapped to the statistical set (Supplementary Figure 11 and Supplementary Table 3). However, the remaining 2,307 experimentally verified VFs from VFDB were not associated with any significant HOGs to HP (Supplementary Table 4), raising the question of why these VFs were not captured by the statistical analysis.

To understand this gap, we assessed the non-significant HOGs corresponding to these 2,307 unassociated VFs, which were in total 3,726 HOGs. Specifically, we assessed their frequency across HP and NHP strains, which consistently fell into one of two distinct patterns: (i) some HOGs were present in only a small number of HP strains (≤ 18) and entirely absent from NHP strains, suggesting potential lineage-specific associations; and (ii) other HOGs were found in both HP and NHP strains with no substantial difference in frequency, indicating broader distribution across HP and NHP bacteria. These findings then suggest that the absence of these VFs from the set of significant HOGs to HP reflects either limited lineage specificity or broad distribution, reducing their statistical association with pathogenicity.

### 3.7 Analysis of pathogenicity domains and novel potential pathogenicity determinants in significant HOGs

Our analysis identified 3,403 significant HOGs to HP that were absent from the VFDB, representing a promising source of potential novel pathogenicity determinants (Supplementary Table 5). To evaluate their biological relevance, we started by annotating their domain composition using HMMER (Finn et al., 2011). Then, we compared the identified domains to those cataloged in the PathFams dataset (Lobb et al., 2021), a comprehensive resource of pathogenic domains in HP bacteria. For comparison, a similar analysis was conducted on a random set of 3,403 non-significant HOGs (Supplementary Tables 6, 7).

A Fisher’s exact test demonstrated that significant HOGs to HP that were not mapped to the experimentally verified VFs from VFDB exhibited a statistically significant enrichment in PathFams domains compared to non-significant HOGs (*p* = 6.145e-11). Among the analyzed domains, significant HOGs contained 746 domains overlapping with PathFams and 2,463 non-overlapping domains, whereas non-significant HOGs contained 386 overlapping and 2,000 non-overlapping domains. In addition, the odds ratio of 1.57 (95% CI: 1.37–1.8), indicates that significant HOGs are 1.57 times more likely to include domains associated with pathogenicity. This result underscores the potential relevance of the significant HOGs to HP to virulence-related functionality.

Examples of significant HOGs which did not overlap with VFDB and may be potential novel pathogenicity determinants for their shared domains with PathFams include Methylcrotonyl-CoA carboxylase and methylglutaconyl-CoA hydratase (Table 3 and previously mentioned in section 3.3); Ribonuclease E inhibitor RraA, which modulates RNase E activity and can potentially affect RNA stability and gene regulation during infection (Chen et al., 2024) (Table 3); and LptD, a key component of lipopolysaccharide biosynthesis which has an indirect role in bacterial virulence (Bertani and Ruiz, 2018).

## 4 Discussion

This study introduces a novel approach to identify potential novel bacterial pathogenicity determinants. The presented analytical framework, highlighted candidate HOGs, and resulting HOGs data may serve as valuable resources for guiding future research. These findings aim to support experimental efforts by prioritizing candidates based on their biological relevance and statistical significance. Our approach redefines bacterial proteomes using HOGs, uncovering potential novel widespread targets that traditional sequence-based methods could overlook. The representativeness of our dataset was demonstrated through comparison with Bartlett et al. (2022), a comprehensive catalog of HP bacteria, and with the FDA-ARGOS wanted organism list. This analysis highlighted the inclusion of key human-associated species, genera, and families, while also incorporating novel taxa not listed in Bartlett et al.

Our study narrows down a vast dataset of 51,745 HOGs to 4,383 HOGs and also a top 20 and 100 HOGs, suggesting prioritized lists of potential novel pathogenicity determinants for future experimental investigations besides already known VFs. Rigorous statistical validation, including Fisher’s exact tests and FDR correction, coupled with a domain-based analysis strengthen the confidence of the pathogenicity associations. In addition, the detection of known VFs also supports the reliability of our approach.

The clustering of the top 100 significant HOGs may reveal critical processes underlying bacterial pathogenicity. The top HOG clusters identified through unsupervised hierarchical clustering within this set reflected distinct functional associations, including fatty acid metabolism, amino acid metabolism, transcriptional regulation, stress response, anaerobic respiration, motility, oxidative stress regulation, signaling, and iron acquisition. For instance, methylcrotonyl-CoA carboxylase and methylglutaconyl-CoA hydratase, exclusively associated with HP strains, may highlight the role of amino acid metabolism in energy production under nutrient-limited conditions. Similarly, the clustering of Lon protease-related proteins alongside fatty acid metabolism regulators suggests metabolic pathways contribute to bacterial resilience and virulence. Clusters emphasizing stress response, antibiotic resistance, and biofilm formation may further underscore adaptive strategies crucial for survival under hostile conditions. The presence of proteins involved in anaerobic respiration and motility structures, such as periplasmic nitrate reductase and flagellar proteins, may highlight adaptive mechanisms for colonization and persistence. Furthermore, clusters associated with oxidative stress regulation, quorum sensing, and cyclic-di-GMP signaling may illustrate sophisticated regulatory networks supporting pathogen adaptability. Lastly, the integration of motility, iron acquisition, and membrane transport proteins may underscore the strategic adaptability of HP bacteria to environmental and host-associated challenges. Overall, the identification of uncharacterized proteins grouped with known VFs highlights potential novel determinants of pathogenicity and targets for therapeutic intervention.

Our results also reveal family-specific patterns of pathogenicity determinants. For instance, within the Enterobacteriaceae family, enzymes such as glucose-1-phosphate thymidylyltransferase, essential for synthesizing nucleotide sugars that contribute to bacterial cell wall, capsule and biofilm formation, were predominantly found in HP strains. This suggests that this protein has important role in maintaining structural integrity and facilitating evasion of host defenses. Within the Corynebacteriaceae family, the consistent presence of SDR enzymes in HP strains suggests a potential novel VF for this group. These enzymes, known to contribute to oxidative stress management, metabolic adaptation and antimicrobial resistance in other bacterial pathogens (Shahri et al., 2024), may play similar roles in this family. Conversely, Streptococcaceae strains exhibited fewer conserved VFs, aligning with the “distributed genome hypothesis,” which posits that Streptococcus pathogens, particularly *S. pneumoniae*, rely on variable VFs tailored to specific ecological niches (Shelyakin et al., 2019; Krzyściak et al., 2013). These findings highlight the importance of both conserved and lineage-specific adaptations in bacterial pathogenicity.

Our HOG-based approach is aimed to complement existing resources such as VFDB by offering a broader perspective on pathogenicity. While VFDB focuses on species-specific, experimentally validated VFs, our method identifies conserved and novel pathogenicity determinants across diverse taxa, including underrepresented and emerging pathogens. By integrating robust statistical validation with domain-level analyses, we detected potential biological relevance of significant HOGs absent from VFDB, which overlap with previously listed HP-related domains. These include indirect yet essential contributors to virulence, such as biosynthetic and stress-response proteins. Therefore, we provide additional insights into conserved and less-direct contributors to virulence, particularly those involved in processes such as host interactions or biosynthetic pathways. HOGs not statistically associated with HP strains while present in VFDB may reflect strain-specific adaptations, as they are either found in a low number of HP strains or in high number in both HP and NHP strains.

However, this study is not without limitations. While statistical associations provide valuable insights, they do not always translate into biological relevance or practical application. Functional assays, such as site-directed mutagenesis or infection models, will be crucial to validate the roles of candidate HOGs in bacterial pathogenicity. Furthermore, improved methods for functional annotation, leveraging dedicated protein databases, are needed to better characterize hypothetical proteins and uncover their potential roles. Finally, incorporating multivariate analyses and advanced classification techniques could enhance the precision of associations between specific HOGs and pathogenicity, offering deeper insights into the molecular mechanisms underlying bacterial infections and their adaptations.

## 6 Conclusion

In conclusion, our study demonstrates the power of phylogenetic-based OA to identify both known and potential novel determinants of bacterial pathogenicity and may constitute a useful resource to guide future works. By leveraging evolutionary relationships and applying rigorous statistical and domain-based validation, we have uncovered potential novel key HOGs that contribute to bacterial pathogenicity. As antibiotic resistance continues to rise, experimental validation of these potential novel widespread pathogenicity factors may be crucial for the development of novel strategies to combat bacterial infections more effectively.

## Data Availability

The datasets presented in this study can be found in online repositories. The names of the repository/repositories and accession number(s) can be found at: The datasets associated with this study are available in Supplementary material or directly via Zenodo under the doi: https://doi.org/10.5281/zenodo.13694408. These include: Supplementary Tables mentioned in this article (Supplementary material); Output file from the OA, named “N0.tsv,” which contains the results of HOGs across different strains, detailing the included proteins for each (Zenodo);Table listing the HOG names assigned for each (Zenodo);Table of the statistically significant HOGs including their names and respective rankings based on the applied criteria (Zenodo).

## Footnotes

1
https://bacspad.altrabio.com/

2
https://zenodo.org/records/13235447
