# Supplementary material for: Identifying potential novel widespread determinants of bacterial pathogenicity using phylogenetic-based orthology analysis
Source: Front Microbiol. 2025 May 1;16:1494490. doi: 10.3389/fmicb.2025.1494490 (PMC12078273; doi:10.3389/fmicb.2025.1494490)
Supplement: Supplementary file 2 [file Data_Sheet_2.pdf]

Supplementary Material

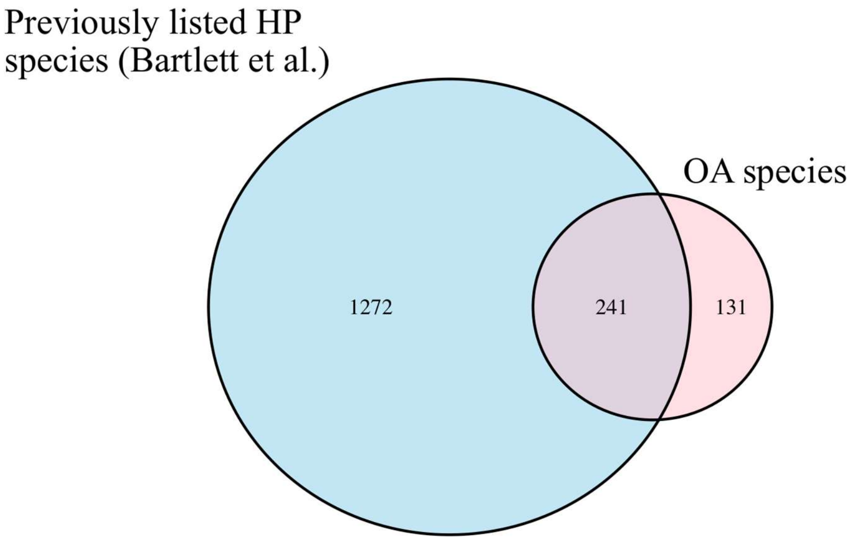

Supplementary Figure 1. Overlap between the number of previously listed species with HP strains and the number of species with HP strains in the orthology analysis (OA) set.

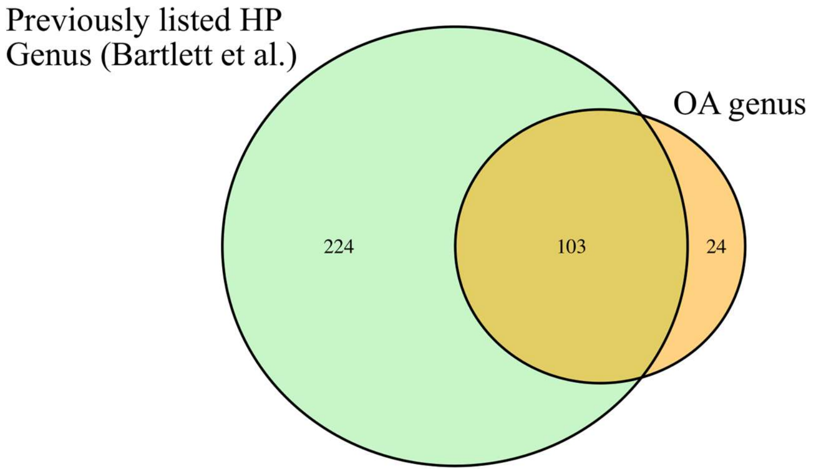

Supplementary Figure 2. Overlap between the number of previously listed genus with HP strains and the number of genera with HP strains in the orthology analysis (OA) set.

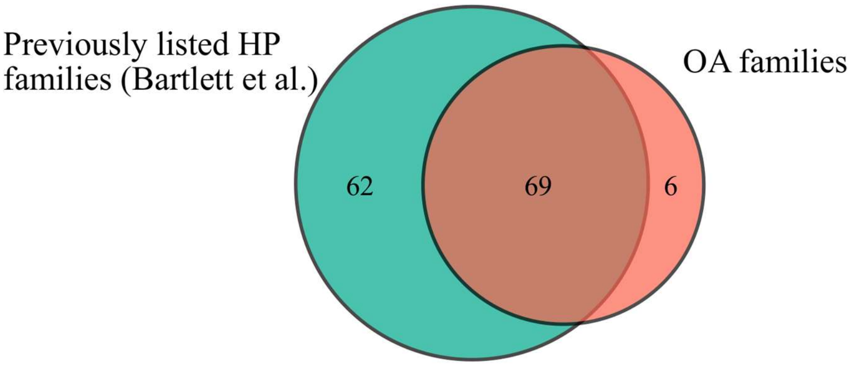

Supplementary Figure 3. Overlap between the number of previously listed families with HP strains and the number of families with HP strains in the orthology analysis (OA) set.

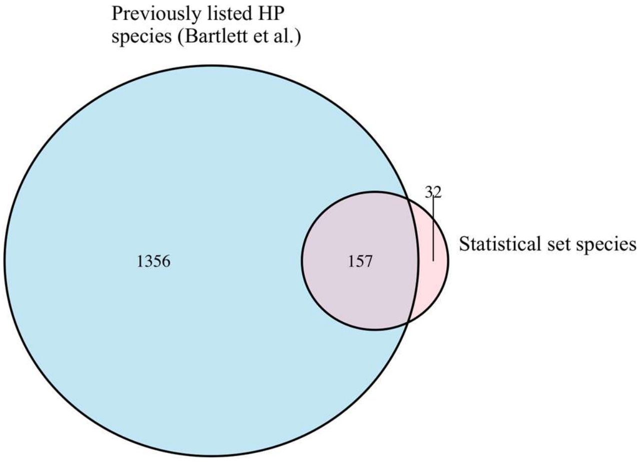

Supplementary Figure 4. Overlap between the number of previously listed species with HP strains and the number of species with HP strains in the statistical set.

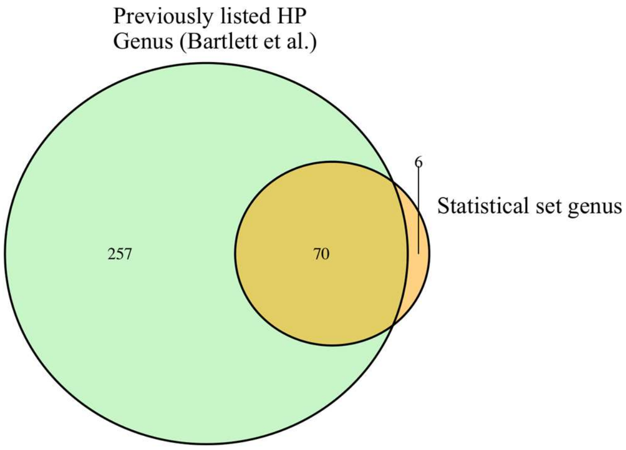

Supplementary Figure 5. Overlap between the number of previously listed genus with HP strains and the number of genera with HP strains in the statistical set.

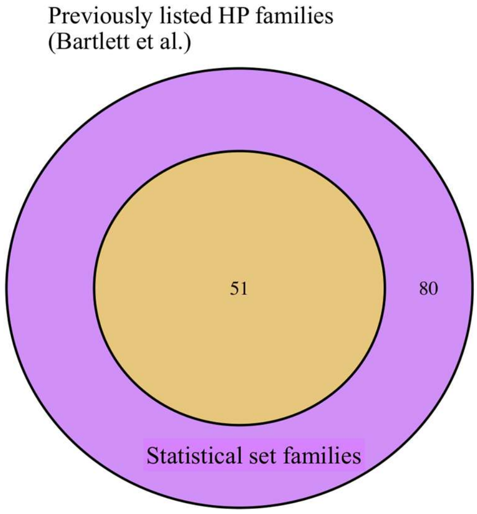

Supplementary Figure 6. Overlap between the number of previously listed families with HP strains and the number of families with HP strains in the statistical set.

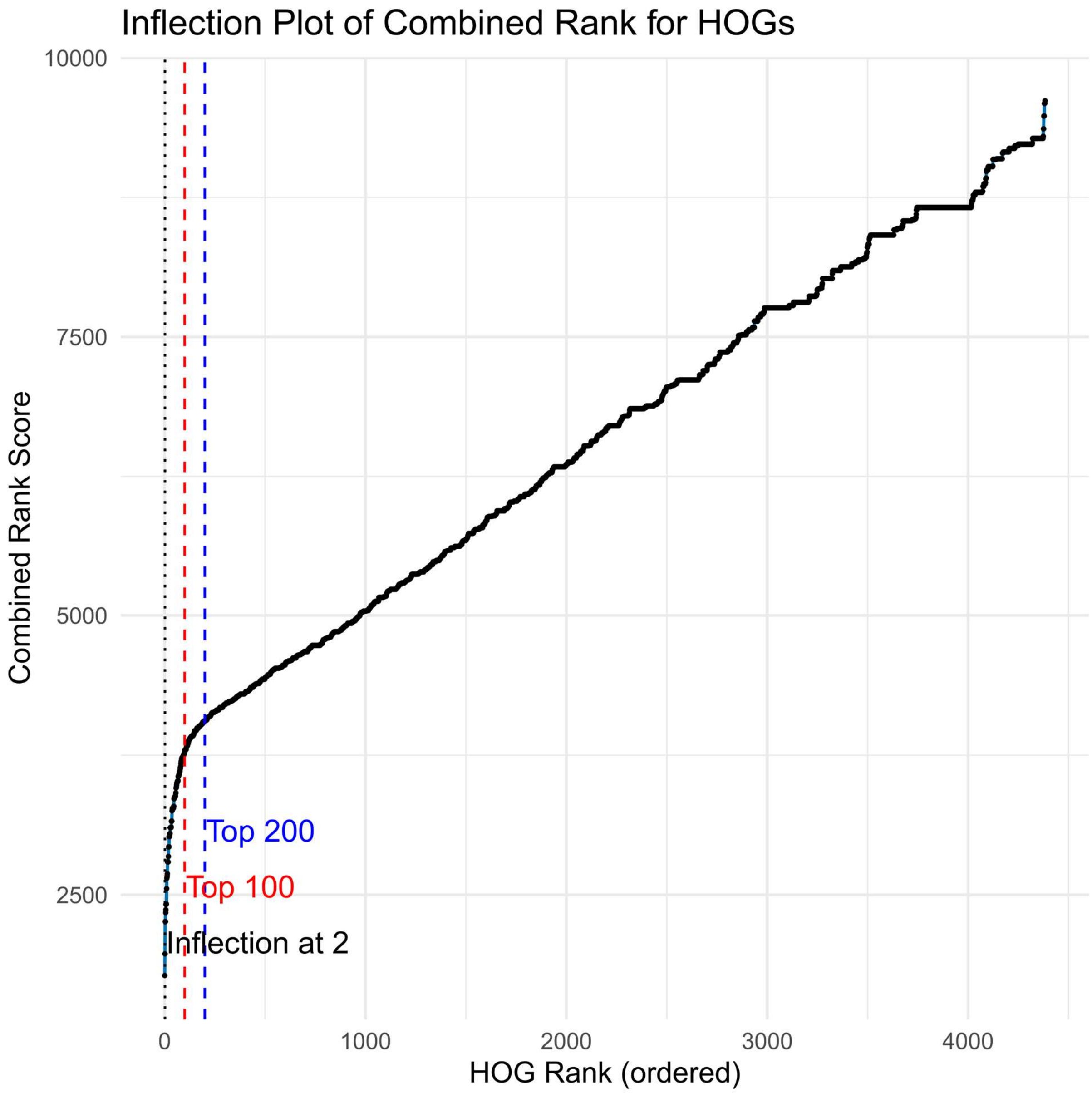

Supplementary Figure 7. Inflection plot of the combined ranking scores for all significant HOGs. Each point corresponds to an HOG, ordered by increasing combined rank score (lower scores indicate higher prioritization). The dashed vertical red and blue lines mark the top 100 and top 200 HOG thresholds, respectively. The dotted black line indicates the inflection point automatically detected using the *kneedle* algorithm. This visualization supports the selection of the top 100 HOGs by showing that they reside where the ranking curve transitions from steep to more gradual increase, which was confirmed by examining the first derivative (slope) of the ranked scores within the top 500 HOGs after the top 5.

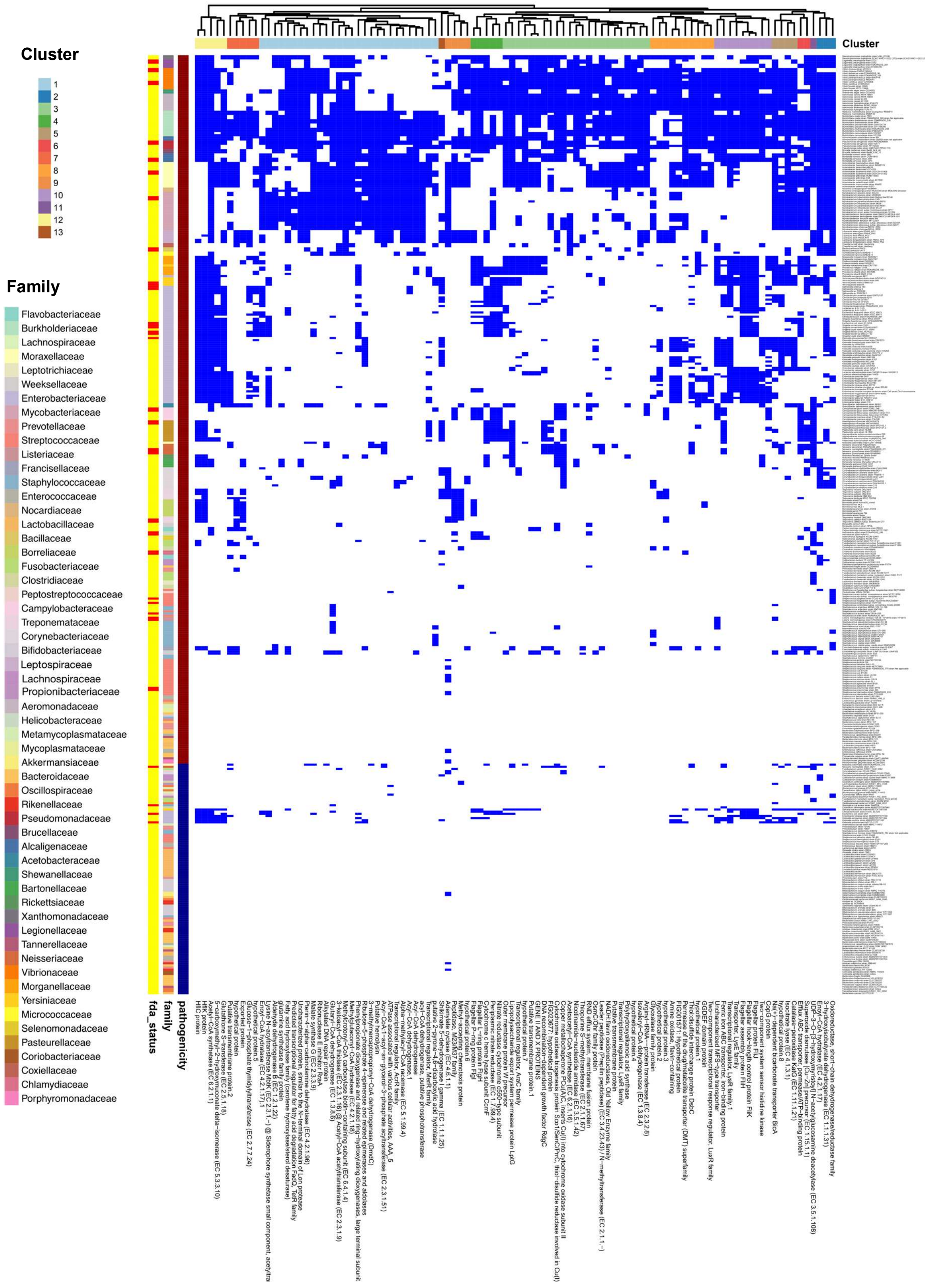

Supplementary Figure 8. Overall view of clustering patterns on the presence (blue)/absence (white) heatmap of the top 100 significant HOGs to HP with strains grouped by pathogenicity labels

(HP/NHP). HOGs are listed below the heatmap, while strain names are displayed on the left. Hierarchical clustering was performed on strains (rows) that contained at least one HOG, as well as on HOGs (columns), using Euclidean distance computed from binary presence (1) / absence (0) values and complete linkage as the clustering method. In this setup, the Euclidean distance between two strains corresponds to the square root of the number of mismatches in their HOG presence/absence profiles. Similarly, the distance between two HOGs reflects how differently they are distributed across strains based on their presence/absence patterns. Distinct clusters resulting from hierarchical clustering of HOGs (1-13) are annotated with different colors above the heatmap, highlighting groups with similar presence/absence patterns. Strains lacking these significant HOGs were manually grouped based on family classification.FDA-status annotations indicate whether a species is listed in the FDA-ARGOS Wanted Organism List (red: yes; yellow: no). Notably, 25 out of 26 species from the FDA-ARGOS Wanted Organism List (Sichtig et al. 2019) were included in the initial data from BacSPaD, providing valuable insights into clinically relevant pathogens. Proteins sharing similar annotations are distinguished by a numerical suffix (e.g., ‘hypothetical protein.1’; ‘hypothetical protein.2’).

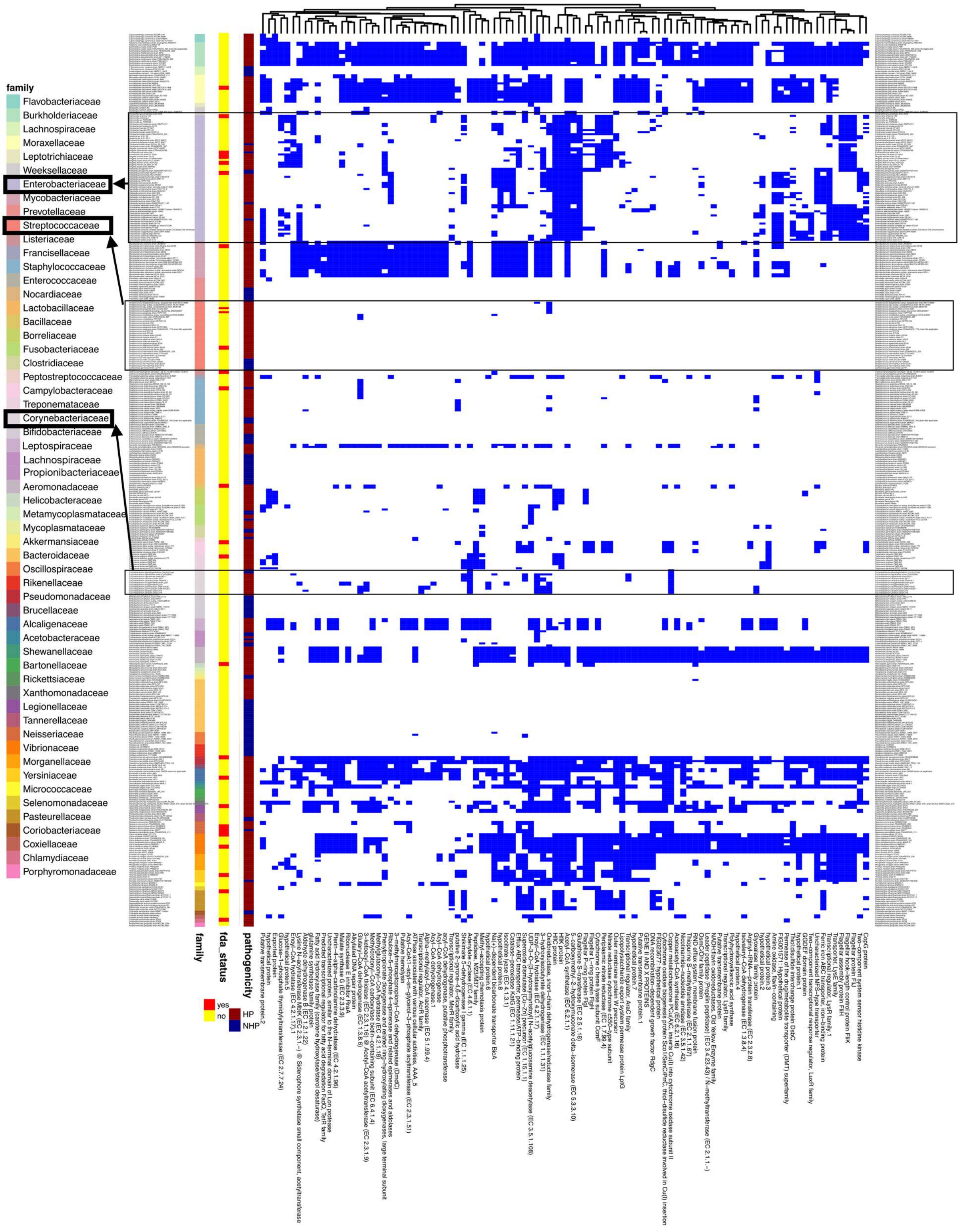

Supplementary Figure 9. Overall view of clustering patterns of the presence (blue)/absence (white) heatmap of the top 100 significant HOGs to HP with strains first grouped by family and then clustered within each family. HOGs are listed below the heatmap, while strain names are displayed on the left. Hierarchical clustering was performed on strains (rows) that contained at least one HOG, as well as on

HOGs (columns), using Euclidean distance computed from binary presence (1) / absence (0) values and complete linkage as the clustering method. In this setup, the Euclidean distance between two strains corresponds to the square root of the number of mismatches in their HOG presence/absence profiles. Similarly, the distance between two HOGs reflects how differently they are distributed across strains based on their presence/absence patterns. Strains lacking these significant HOGs were manually ordered by their pathogenicity label (HP/NHP) within each family. FDA-status annotations indicate whether a species is listed in the FDA-ARGOS Wanted Organism List (red: yes; yellow: no). Proteins sharing similar annotations are distinguished by a numerical suffix (e.g., ‘hypothetical protein.1’; ‘hypothetical protein.2’).

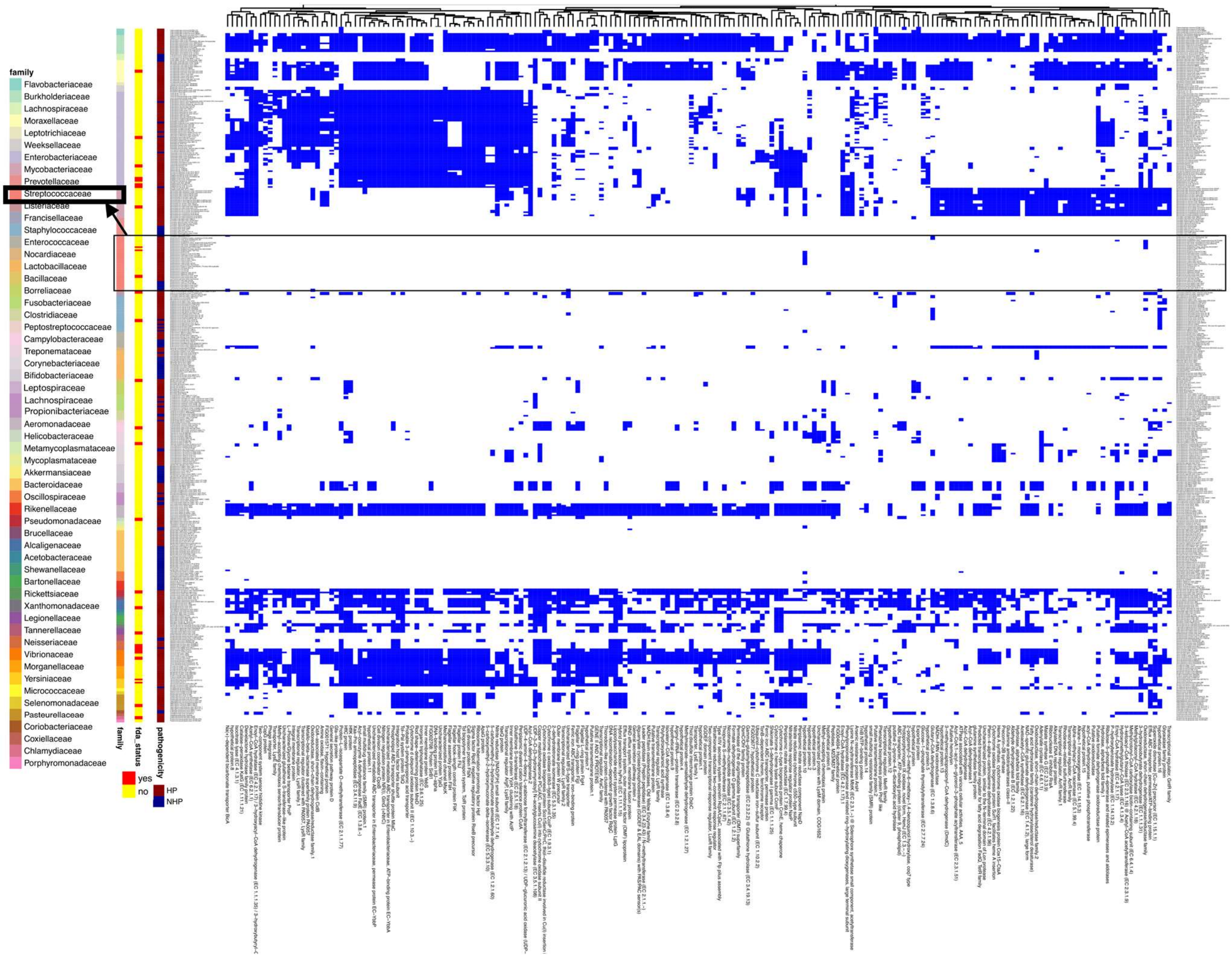

Supplementary Figure 10. Overall view of clustering patterns presence (blue)/absence (white) heatmap of the top 200 significant HOGs to HP, with strains grouped by family and then clustered within each family. HOGs are listed below the heatmap, while strain names are displayed on the left. Hierarchical clustering was performed on strains (rows) that contained at least one HOG, as well as on HOGs (columns), using Euclidean distance computed from binary presence (1) / absence (0) values and complete linkage as the clustering method. In this setup, the Euclidean distance between two strains

corresponds to the square root of the number of mismatches in their HOG presence/absence profiles. Similarly, the distance between two HOGs reflects how differently they are distributed across strains based on their presence/absence patterns. Strains lacking these significant HOGs were manually ordered by their pathogenicity label (HP/NHP) within each family. FDA-status annotations indicate whether a species is listed in the FDA-ARGOS Wanted Organism List (red: yes; yellow: no). Proteins sharing similar annotations are distinguished by a numerical suffix (e.g., 'hypothetical protein.1'; 'hypothetical protein.2').

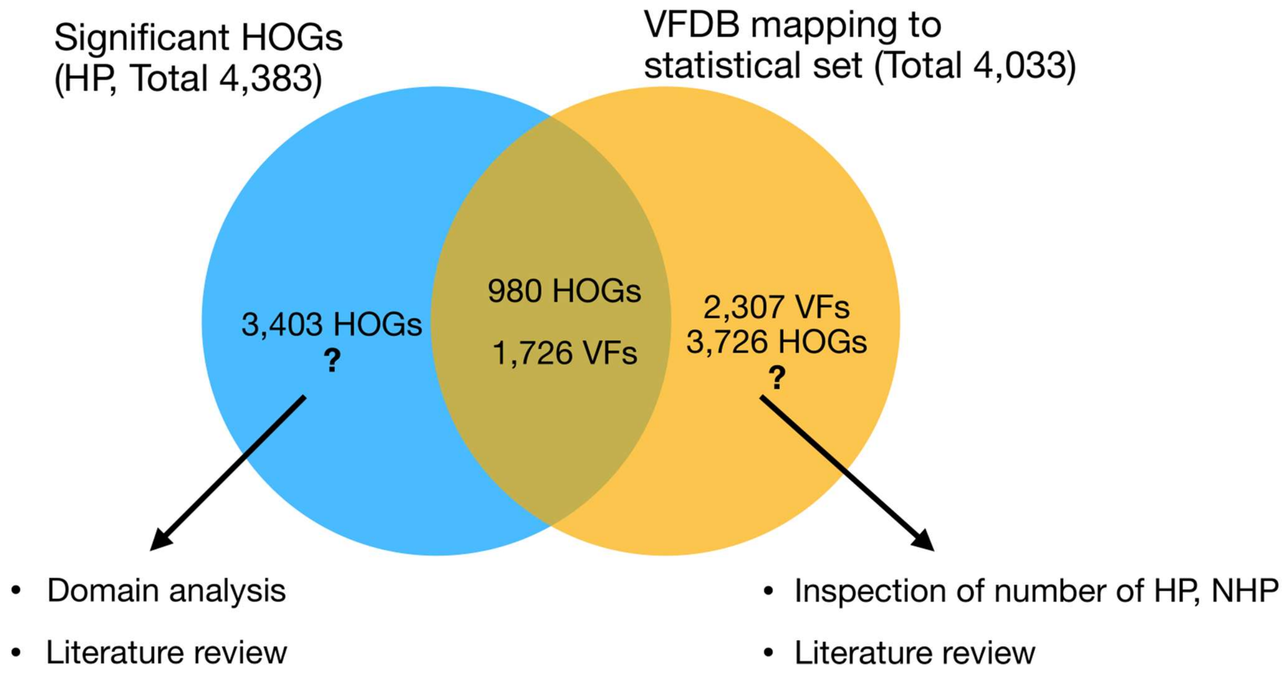

Supplementary Figure 11. Overlap between the number of statistically significant hierarchical orthogroups (HOGs) to pathogenic to humans (HP) strains and the number of experimentally validated virulence factors (VFs) from Virulence Factor Database (VFDB) present in the statistical test set. For the 3,403 significant HOGs not in VFDB (left), an analysis of pathogenicity domains and novel potential pathogenicity determinants was followed. For the 3,726 HOGs mapping to the 2,307 VFs that did not map to the significant HOGs (right), both an inspection of their number of HP and NHP strains was followed. Both of these steps were complemented with literature review to assess functional annotations and their relevance for bacterial pathogenicity.
